# Prognosis and postoperative genital function of function-preservative surgery of pelvic autonomic nerve preservation for male rectal cancer patients

**DOI:** 10.1186/s12893-016-0127-4

**Published:** 2016-03-13

**Authors:** Zhihua Liu, Meijin Huang, Liang Kang, Lei Wang, Ping Lan, Ji Cui, Jianping Wang

**Affiliations:** Gastrointestinal Institute of Sun Yat-Sen University, Department of Colorectal Surgery, the Sixth Affiliated Hospital of Sun Yat-Sen University (Guangdong Gastrointestinal Hospital), 26 Yuancun Erheng Road, Guangzhou, Guangdong 510655 People’s Republic of China; Department of Gastrointestinal Surgery, the First Affiliated Hospital of Sun Yat-Sen University, Guangzhou, Guangdong China

**Keywords:** Rectal cancer, Total mesorectal excision, Pelvic autonomic nerve preservation, Genital function, Survival rate

## Abstract

**Background:**

To retrospectively evaluate postoperative genital function, local recurrence rate and survival rate after total mesorectal excision (TME) combined with or without pelvic autonomic nerve preservation (PANP) in male patients with rectal cancer.

**Methods:**

A total of 953 male patients with rectal cancer after TME (518 patients received TME combined with PANP [PANP group] and 434patients received TME alone [TME group]) were included. Assessments of postoperative genital function, local recurrence rate, and 5 year survival rate were collected.

**Results:**

Rate of erection dysfunction in PANP group (41.9 %) was significantly lower than that in TME group (76.7 %, *P* < 0.05). Rate of ejaculation dysfunction in PANP group (42.5 %) was also significantly lower than that in TME group (67.3 %, *P* < 0.05). Local recurrence rate (*P* = 0.66) and survival rate (*P* = 0.26) did not differ between the two groups. For patients with preoperative obstruction, local recurrence rate was significantly higher (*P* = 0.01) and survival rate significantly lower (*P* = 0.03) in PANP group.

**Conclusions:**

PANP surgery has significant advantage with respect to preservation of genital function and should be recommended as surgical treatment for rectal cancer patients. However, PANP surgery should be considered with caution in patients with preoperative obstruction in view of the poorer long-term outcomes in these patients.

## Background

It has been estimated that over one million patients are diagnosed with colorectal cancer (CRC) every year, along with 500,000 related deaths in the world [1, 2]. Mortality and morbidity of CRC have been rising in recent years [3] and CRC has become the third most common type and the fourth most common mortality of malignant carcinoma all over the world [4]. The overall incidence of CRC is up to 5 % in general population and the overall 5 year survival rate is less than 60 % in the world [5]. It has also been estimated that the lifetime risk of developing CRC was up to 6 % [6]. In developing countries like China, especially in major cities such as Guangzhou, Beijin, and Shanghai, as lifestyle alters,burden of CRC has rapidly increased [7, 8]. Moreover, the epidemiological characteristics of CRC in Chinese patients, known as “three high and one low” [9, 10], present as a severe threat [11]. These include high proportion of rectal cancer (RC, about 60 %), high proportion of distal RC (about 65–70 %), and high proportion of young patients (10–15 % of patients less than 30 years old), and low early diagnostic rate (less than 10 %) [12]. Low position of RC tumor increase ssurgical difficulty andyoung age raises the issue of genital function preservation.

Severe genital dysfunction associated with the injury of autonomic nerve is a common complication of middle and distal RC surgery. It has been suggested that total mesorectal excision (TME) combined with pelvic autonomic nerve preservation (PANP) for the treatment of RC could lower the rate of postoperative genital dysfunction [13]. However, whether PANP can retain the radical cure effect is still in debate. In this study, we aimed to compare the radical cure effect of TME combined with and without PANP as surgical treatments for RC and to compare postoperative genital function, local recurrence, and survival rate between the two treatments.

## Methods

### Patients

All male patientsaged between 18 and 60 years old who had a diagnosis of RC and underwent radical proctectomy surgery in the Sixth Affiliated Hospital of Sun Yat-sen University between October 1997 and December 2013 were reviewed for inclusion in this study. Diagnosis of RC was established based on comprehensive review of medical history, physical examination, three-phase contrast-enhanced computed tomography (CT) findings, magnetic resonance imaging (MRI) findings, colonoscopy features, and surgical findings as previously described [14]. Inclusion criteria were: 1) CT scan, MRI or colonoscopy suggesting RC; 2) adenocarcinoma; and 3) no metastasis. Exclusion criteria were: 1) genital dysfunction after neoadjuvant chemo-radiotherapy and before operation; 2) symptoms suggesting bowel perforation; 3) septic symptoms; 4) patients at Dukes B and C stages who rejected neoadjuvant chemo-radiotherapy; 5) adjacent small bowel involvement. This study was approved by the Ethics Committee of Sun Yat-sen University and was conducted in accordance to the Declaration of Helsinki. All participants provided written informed consent.

### Surgical technique

One day before the surgery, patients were given Soffodex, which contained 2.4 g of monobasic sodium phosphate and0.9 g of dibasic sodium phosphate, for mechanical bowel preparation. Preoperative carbohydrate loading was not performed. As described previously [15], open surgical approach of proctectomy was performed using the midline incision. During the surgery, oral bowel preparation and nasogastric tubes were planted as routine use. Patients underwent radical proctectomy with high ligation of the inferior mesenteric vessels and TME. The rectum was mobilized with an ultrasonic scalpel dissecting between the visceral and parietal pelvic fascia. Laparoscopic rectomy was performed using a 5-port technique and a transverse incision of about 5 cm for extraction of the specimen. Patients were placed in the modified lithotomy position [16] with the surgeon standing on the right side of the patient. Clips or vascular staplers were used form obilization, isolation and ligation for the main vessels from medial to lateral of rectal tissues [17]. TME was performed using monopolar diathermy and ultrasonic scalpel [15, 18]. A wound protector was used to protect of the sample extraction site, and anastomoses were performed intracorporeally. A loop ileostomy was routinely planted for mid and low rectal anastomoses for postoperative recovery of anastomotic stoma. The PANP surgery had three steps, including revealing sacral nerve plexus and hypogastric nerve (Ejaculation nerve), preservation of the integrity of at least one-side parietal pelvic fascia and preservation of the envelop of seminal vesicle (Denonvilliers facia). We classified PANP into four types. Type I referred to complete preservation of the pelvic autonomic nerve. For type II, the hypogastric nerve was cut off but the pelvic plexus was preserved. For type III, the hypogastric nerve and unilateral pelvic plexus were cut off but the other lateral pelvic plexus was preserved. For type IV, the hypogastric nerve and bilateral pelvic plexuses were completely cut off. Selection of types of PANP was according to the tumor position and depth of invasion.

Postoperative management included minimal use of drains, early unrestricted postoperative oral intake of fluids and light diet, early mobilization, selected use of epidural catheters, and restricted use of narcotics [15]. Postoperative pain was managed by selective epidural catheters and patient controlled analgesia. Patients were discharged when criteria were met [19]. Regime of fluorouracil combined with calcium folinate were considered as the first-line treatment of chemotherapy 1 month after surgery. Neoadjuvant chemo-radiotherapy were recommended for patients at Dukes B and C stages.

### Follow-up

Follow-up assessments were performed by one commissioner (Biying Yi) and three surgeons (Meijin Huang, Chao Li, and Xingwei Zhang), through clinic appointments, home visits, and/or letters or phone calls. Assessments included physical examination, haematological and biochemical examinations, serum carcinoembryonic antigen level, chest X-ray, and abdominal and/or pelvic CT scan every 3 months during the first year, every 6 months during the subsequent 2 years, and then once a year afterwards [14]. The follow-up end point was February 2014.

Five-year survival rate was extracted from the database of CRC patients in the Sixth Affiliated Hospital of Sun Yat-sen University. Death occurring within 30 days after the surgery was referred as surgical complication and excluded from the survival analysis [14]. All causes of death were included in the study. Curative procedure referred to complete resection of tumorwith no local or distant residual malignancy and palliative procedure referred to recurrent local malignancy or metastasis [14]. Local recurrence referred to recurrence near the anastoma or pelvic recurrence. Postoperative genital function was evaluated as erection function and ejaculation function before discharge of patients and 6 months after surgery. Erection function was classified as: I, normal and complete erection, no difference from preoperative status; II, decrease of erection function, partial erection, decrease of flintiness after erection; III, no erection or loss of erection. Ejaculation function was classified as: I, normal ejaculation, including decreased ejaculation but with orgasm; II, retrograde ejaculation, with ejaculation dysfunction; III, no ejaculation.

### Statistical analysis

Statistical analysis was performed using the SPSS for Windows Version17.0 (SPSS, Chicago, IL, USA). Demographic data between the two groups were compared using Student’s *t*-test for continuous data and χ^2^ test for categorical data. The survival rate was calculated by the Kaplan-Meier method and difference in survival was compared by log-rank test. All hypotheses were two-tailed and p-value less than 0.05 was considered statistical significant.

## Results

### Patient characteristics

A total of 952 patients were included in this study. Among these patients, 434 patients received TME surgery (control group) and 518 patients received TME combined with PANP surgery (PANP group). There was no significant between-group difference in baseline characteristics, including age, body mass index, presence of obstruction, distance from the lower edge of the tumour to anus, size of the tumour, use of laparoscopy, Dukes stages, and histological grades (Table 1).

**Table 1:** Baseline patient characteristics of study patients

Variables	PANP (*n* = 518)	Control (*n* = 434)	χ^2^/T value	P value
Age, years	58.61 ± 14.29	57.47 ± 13.51	1.26	0.21
Body mass index, kg/m^2^	22.16 ± 3.70	22.52 ± 3.55	1.53	0.13
Obstruction^a^	51 (10.9 %)	26 (6.4 %)	4.72	0.03
Distance from the lower edge of the tumour to anus (cm)	6.26 ± 2.86	6.01 ± 2.67	1.38	0.17
Tumour size (cm)			0.06	0.87
≤5	101 (19.5 %)	82 (18.9 %)		
>5	417 (80.5 %)	352 (81.1 %)		
Laparoscopy	262 (50.6 %)	240 (55.3 %)	2.11	0.15
Dukes stage^b^			1.97	0.16
A	106 (20.9 %)	88 (20.3 %)		
B	214 (41.3 %)	160 (36.9 %)		
C	113 (22.3 %)	114 (26.3 %)		
D	73 (14.4 %)	72 (16.6 %)		
Histological grade			2.55	0.28
Well differentiated	184 (35.5 %)	136 (31.3 %)		
Moderately differentiated	232 (44.8 %)	216 (49.8 %)		
Poorly differentiated	102 (19.7 %)	82 (18.9 %)		
Surgical approach			1.77	0.19
Low anterior resection	130 (25.1 %)	93 (21.4 %)		
Abdominoperineal resection	388 (74.9 %)	341 (78.6 %)		

Results are presented as mean ± SD or number (%). *PANP* pelvic autonomic nerve preservation

^a^Available in 467 patients in PANP group and 408 patients in control group

^b^Available in 506 patients in PANP group and 434 patients in control group

### Postoperative genital function

Table 2 shows postoperative genital function before discharge and 6 month after surgery. Rate of erection dysfunction (classified as II or III) in PANP group was 41.9 %, compared with 76.7 % in control group (*P* < 0.001). Rate of ejaculation dysfunction (classified as II or III) in PANP group was 42.5 %, compared with 67.3 % in control group (*P* < 0.001). Rate of erectionor ejaculation dysfunction remained significantly lower 6 months and 1 year after surgery in PANP group.

**Table 2:** Short-term and long-term outcomes after proctectomy

	Surgical approach		χ^2^/t value	*p* value
	PANP	Control		
Before discharge Erection dysfunction			120.53	<0.001
I	301	101		
II	121	211		
III	96	122		
Ejaculation dysfunction			240.53	<0.001
I	298	142		
II	124	186		
III	96	106		
Half a year after surgery				
Erection dysfunction			122.54	<0.001
I	316	111		
II	112	203		
III	90	120		
Ejaculation dysfunction			61.52	<0.001
I	306	148		
II	120	182		
III	92	104		
One year after surgery				
Erection dysfunction			124.10	<0.001
I	319	116		
II	110	206		
III	89	122		
Ejaculation dysfunction			60.42	<0.001
I	310	152		
II	119	180		
III	89	102		
Local recurrence	50/468	38/396	0.23	0.66

Results are presented as number (percentage). *PANP* pelvic autonomic nerve preservation

### Long-term outcomes

**Fig. 1:**
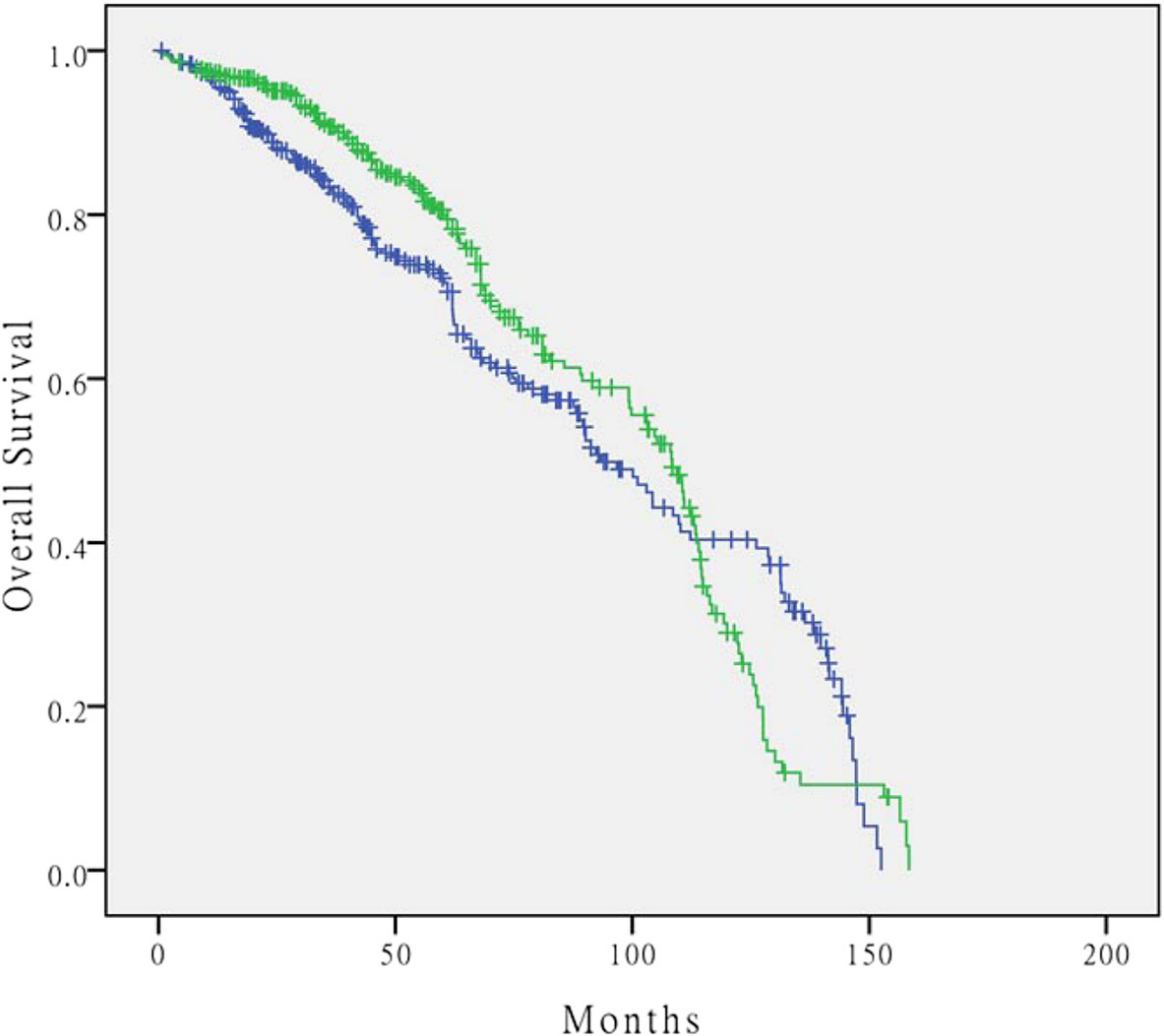
Kaplan-Meier curve showing overall survival rates for rectal cancer patients who underwent proctectomy of PANP surgery or TME only surgery. No significant difference in survival rates was found between the two groups (*P* = 0.26). The green line represents PANP group and blue represents TME only group. PANP: pelvic autonomic nerve preservation; TME: total mesorectal excision

The overall local recurrence rate in PANP group was 9.7 %, compared with 8.8 % in control group, with no significant between-group difference (*P* = 0.66, Table 2). Median survival time in control and PANP groups was 93.4 and 108.3 months, respectively, with no significant between-group difference (*P* = 0.26, Fig. 1).

### Influence of obstruction in long-term outcomes for PANP surgery

**Fig. 2:**
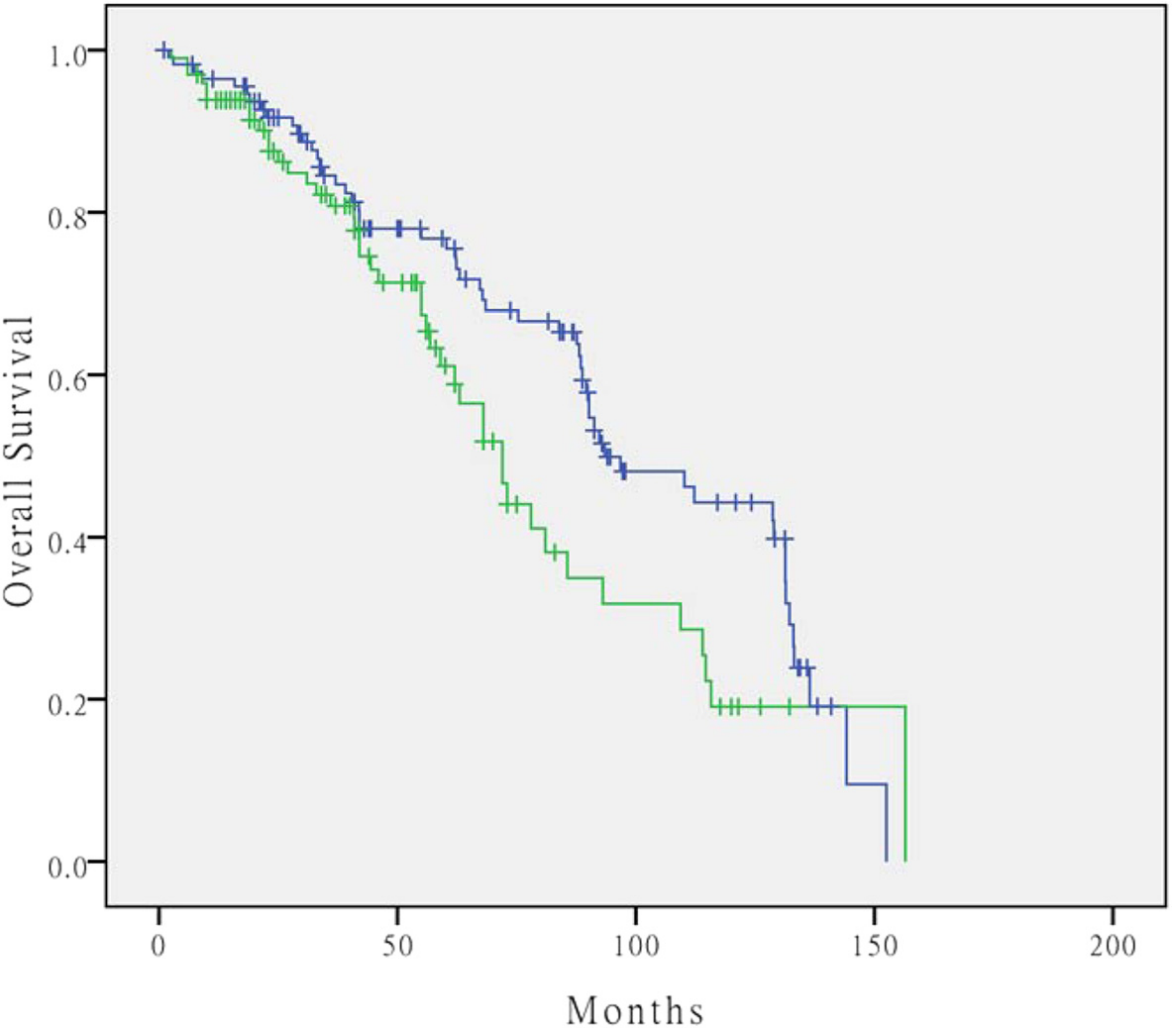
Kaplan-Meier curve showing overall survival rates for rectal cancer patients with preoperative obstruction who underwent proctectomy of PANP surgery or TME only surgery. Survival of patients with obstruction in PANP group was significantly lower than those in control group (*P* = 0.032). The green line represents PANP group and the blue represents TME onlygroup. PANP: pelvic autonomic nerve preservation; TME: total mesorectal excision

Two hundreds and twelve patients (114 patients in control group and 98 in PANP group) had preoperative obstruction. Local recurrence rate in patients with preoperative obstruction in PANP group (16.3 %) was significantly higher than that in control group (5.3 %) (*P* = 0.012). Survival analysis also showed significantly lower survival in patients with preoperative obstruction in PANP group (*P* = 0.032, Fig. 2).

## Discussion

Traditional TME requires routine resection of mesorectum, including fat, lymph nodes, and fibrous tissues [20], which increases the risk of pelvic autonomic nerve injury and genital dysfunction [21]. Maurer et al. reported that rate of urinary dysfunction for TME surgery was similar to that for conventional rectal cancer surgery, but rate of genital dysfunction was lower for conventional surgery [22]. Our results demonstrated significant advantage of PANP surgery over TME alone surgery with regard to preservation of genital dysfunction, including erection and ejaculation function. The common reasons for pelvic autonomic nerve injury include a lack of anatomic knowledge and aggressive dissection. PANP surgery requires more precise anatomic dissection and knowledge of the pelvic anatomy.

It has been reported that rate of postoperative genital dysfunction after rectal cancer surgery was from 40 to 100 % [23]. Such large variation might be due to differences in characteristics of the study cohorts [24, 25]. Age could be an important factor for postoperative genital function. Most studies included patients less than 60 years old. Preoperative genital dysfunction could be common among old people and genital function of these patients could be further damaged after the surgery [26, 27].

United States (US) and European countries hold different opinions with regard to genital function preservation associated with RC surgery. US recommends TME combined with PANP surgery, while European countries emphasize the lateral lymph nodes resection. In China, the surgical technique of TME combined with PANP has received increasing attention. Our results indicated that postoperative local recurrence rate and 5 year survival rate did not differ significantly between PANP group and control group, indicating that PANP technique has comparable long-term outcomes. Similar results have been reported by Shirouzu et al. in an analysis of 403 RC patients who received TME with or without PANP. Their results showed that the local recurrence rate, metastasis rate, and survival rate in PANP group were not inferior to TME alone group [28]. On the other hand, the PANP surgery could effectively preserve the postoperative genital function, which could lead to improved quality-of-life in these patients, an important issue for younger patients. In addition, PANP surgery induces less injury to genital vessels and less blood loss, hospital stay could be significantly shortened compared with control group. These findings have significant clinical relevance for regions in which RC occurs at a younger age and hence, PANP should be the preferred surgical technique.

Previous studies showed that obstruction was a risk factor for postoperative outcomes for RC patients [14, 29]. Therefore, we further analysed the influence of obstruction on local recurrence rate and 5 year survival rate. We found that for patients with preoperative obstruction, local recurrence rate and 5 year survival rate were significantly poorer in patients who received PANP than those who received TME only. This could be related to surgical difficulty, risk of potential tumour residual, and lower rate of curative resection [14, 29]. These findings indicated that for RC patients with preoperative obstruction, TME combined with PANP surgery should be considered with caution.

Furthermore, for patients with risk factors such as tumour invasion out of the intestinal wall and tumour with lymph metastasis, balance should be achieved between radical resection and preservation of pelvic autonomic nerve. In majorities of cases, radical resection should be the priority and PANP could be used considering the depth of invasion of the tumour. For patients of Dukes A stage, complete preservation of the pelvic autonomic nerve should be preferred. For those of Ducks B or Ducks C stages with tumour upon the peritoneal reflection, it would be preferable to cut off the hypogastric nerve and retain the pelvic plexus. For those of ducks B or C stages, cutting off the hypogastric nerve and unilateral pelvic plexus, while retaining the other lateral pelvic plexus is preferred. For patients with lateral lymph nodes metastases, cutting off the hypogastric nerve and bilateral pelvic plexuses completely is recommended.

One limitation of our study is that it was a retrospective analysis. Further studies using a randomized controlled trial design should be performed to confirm our findings. Another limitation is that the percentage of patients with Duke A and B stages are slightly higher in PANP group than that in control group, which could present as a potential confounder. However such difference was not statistically significant.

## Conclusion

In conclusion, our results showed that PANP surgery has significant advantage with respect to preservation of genital function and should be recommended as surgical treatment for RC patients. However, PANP surgery should be considered with caution in patients with preoperative obstruction in view of the slightly poorer long-term outcomes in these patients.

## Abbreviation

CRC: Colorectal cancer
CT: Computed tomography
MRI: Magnetic resonance imaging
PANP: Pelvic autonomic nerve preservation
RC: Rectal cancer
TME: Total mesorectal excision
